# MicroRNA-7 Targets the KLF4 Gene to Regulate the Proliferation and Differentiation of Chicken Primary Myoblasts

**DOI:** 10.3389/fgene.2020.00842

**Published:** 2020-09-18

**Authors:** Genxi Zhang, Fuxiang Chen, Pengfei Wu, TingTing Li, Mingliang He, Xuemei Yin, Huiqiang Shi, Yanjun Duan, Tao Zhang, Jinyu Wang, Kaizhou Xie, Guojun Dai

**Affiliations:** ^1^College of Animal Science and Technology, Yangzhou University, Yangzhou, China; ^2^Joint International Research Laboratory of Agriculture and Agri-Product Safety, Yangzhou University, Yangzhou, China; ^3^Jiangsu Jinghai Poultry Group Co., Ltd., Nantong, China

**Keywords:** primary myoblasts, miR-7, KLF4, proliferation, differentiation

## Abstract

The proliferation and differentiation of chicken primary myoblasts (CPMs) play an important role in the development of skeletal muscle. In our previous research, RNA-seq analysis showed that microRNA-7 (miR-7) was relatively highly expressed in the proliferation phase of CPMs, but its expression level decreased significantly after CPM_S_-induced differentiation. Meanwhile, the mechanism by which the miR-7 regulates the proliferation and differentiation of CPMs is still unknown. In this study, we found that the expression levels of miR-7 and the Krüppel-like factor 4 (KLF4) gene were negatively correlated during the embryonic phase, and *in vitro* induced differentiation. A dual-luciferase assay and a rescue experiment show that there is a target relationship between miR-7 and the KLF4 gene. Meanwhile, the results show that overexpression of miR-7 inhibited the proliferation and differentiation of CPMs, while inhibition of miR-7 had the opposite effects. Furthermore, overexpression of the KLF4 gene was found to significantly promote the proliferation and differentiation of CPMs. Conversely, inhibition of the KLF4 gene was able to significantly decrease the proliferation and differentiation of CPMs. Our results demonstrate, for the first time, that miR-7 inhibits the proliferation and differentiation of myoblasts by targeting the KLF4 gene in chicken primary myoblasts.

## Introduction

The embryonic period is a critical period for the growth of broilers, and the number of muscle fibers is also mostly determined during this period. Muscle fibers are an important part of muscles and it has been found that muscle fiber density affects muscle protein content. The satellite cells in muscles are called myoblasts, and in recent years, myoblasts have been identified as one of the key components in muscle development and endogenous repair (Relaix and Zammit, 2012; Yin et al., 2013), and account for about 2–10% of the total number of myonuclei (Dumont et al., 2015). As a precursor of skeletal muscle fibers, myoblasts first fuse into myotubes after a series of proliferation, and finally differentiate into mature muscle fibers (Wang, 2017; Li et al., 2018a; Wang et al., 2018a). The proliferation of myoblasts and their differentiation into myotubes largely determines the number of muscle fibers after birth.

MicroRNAs (miRNAs) are a class of small non-coding RNAs with a length of about 23 bases. miRNAs degrade or inhibit the expression of target genes by pairing with the 3′untranslated region (3′UTR) of the target mRNAs, thereby regulating the expression of the target genes post-transcriptionally (Pasquinelli and Ruvkun, 2002; Sun et al., 2017). These small non-coding RNAs are critical for the regulation of intracellular translational processes and play a key role in several cellular life activities, including differentiation, proliferation, and signal transduction (Stefani and Slack, 2008; Ebert and Sharp, 2012; Gurtan et al., 2012). Some miRNAs have been reported to affect the proliferation and differentiation of chicken myoblasts. Wang et al. (2018b) found that miR-205a is able to repress the proliferation and to promote the differentiation of chicken myoblasts, while microRNA-664-5p is able to promote the proliferation and to inhibit the differentiation of chicken myoblasts (Cai et al., 2018b). MiR-7 is a well-known tumor suppressor that is involved in the development of different types of human tumors and in the treatment of tumor diseases (Gu et al., 2015). Zeng et al. (2018) showed that the overexpression of miR-7 can significantly inhibit the proliferation, migration, and invasion of colorectal cancer cells (CRC), as well as induce the apoptosis of CRC. In our previous research (Li, 2018), RNA-seq analysis showed that miR-7 is relative highly expressed during the proliferation of CPMs, but its expression level decreased significantly after the CPM_S_-induced differentiation. However, the way in which miR-7 regulates the proliferation and differentiation of CPMs remains unknown.

Combined with miR-7 target prediction and the RNA-Seq results, we identified Krüppel-like factor 4 (KLF4) as the candidate target gene of miR-7 in chicken myoblasts. The KLF4 gene, also known as the epidermal zinc finger factor, is an evolutionarily conserved zinc finger-containing transcription factor that regulates cell growth, proliferation, and differentiation (Ghaleb and Yang, 2017). Miao et al. (2017) found that miR-346 regulates the proliferation, differentiation, and apoptosis of neural stem cells (NSCs) by targeting KLF4, which is a core transcriptional factor determining the fate of NSCs. By deleting the KLF4 gene in mouse neural precursor cells, Moon et al. (2018) found that the KLF4 gene not only functions as a transcription factor, but also promotes the formation of nerve cells. Antin et al. (2010) reported the expression of chicken KLF4 during the first 5 days of embryonic development, and deduced that KLF4 has multiple functions during the early stages of embryonic development. However, the way in which the KLF4 gene regulates the proliferation and differentiation of CPMs remains unclear.

The Jinghai Yellow chicken is a national yellow-feathered broiler breed. It has the characteristics of small size and delicious meat, but it grows slowly compared to white-feathered broilers (Zhang et al., 2015). In this study, the main purpose was to investigate the effect of miR-7 on the proliferation and differentiation of CPMs, and to elucidate the regulatory mechanism associated with these processes. It is expected that the results will enrich the molecular mechanism of miRNAs regarding the growth and development of the skeletal muscle of chickens, and also provide a theoretical basis for the molecular breeding of chickens.

## Materials and Methods

### Experimental Animals and Tissues

Jinghai Yellow chickens were hatched in the Genetic Breeding and Reproduction Laboratory of Jiangsu Province. Four female Jinghai Yellow chickens from the same incubator at the age of 12 embryonic days (E12), E14, E16, E18, E20, and at 1-day-old (day 1) (the sex was judged by the development of the gonad) were randomly selected. The chest and leg muscle tissues from the five embryonic stages were collected. For the 1-day-old female chickens, a total of nine types of tissue (i.e., the heart, liver, spleen, lung, kidney, intestine, gizzard, chest muscle, and leg muscle) were collected. The tissues were soaked in RNA preservation solution (Vazyme, Nanjing, China) and then stored at –20°C.

### Cell Culture

The culture of 293T cells: The 293T cell line of human renal epithelial cells was cultured in Dulbecco’s Modified Eagle medium (DMEM)–High Glucose (Sigma-Aldrich, St. Louis, MO, United States), supplemented with 10% (v/v) fetal bovine serum (FBS; Biological Industries, Kibbutz Beit Haemek, Israel) and 1% antibiotic–antimycotic (Solarbio, Beijing, China).

The isolation and culture of chicken primary myoblasts (CPMs): CPMs were extracted from the leg muscle of 12-day-old chicken embryos. The bones and blood clots in the muscles were separated, and the muscles were cut into meat sludge in a 100 mm sterile petri dish using medical scissors. D-Hank’s solution was added to the petri dish and then transferred into a 15 ml centrifuge tube. After standing for 5 min, the supernatant was discarded (to remove the fascia and blood cells), and then two volumes of type I collagenase digestive fluid were added. The suspension was digested with collagenase I for 20 min at 37°C, and then complete medium with 20% FBS was added to quench the digestion. The sieved cells were collected by centrifugation at 1200 × *g*. On this basis, differential adherence was performed three times to remove the fibroblasts. The CPMs were then cultured in complete medium with 20% FBS. The culture dishes were incubated at 37°C in a humidified atmosphere with 5% CO2 (incubator: Binder, Tuttlingen, Germany). When the cell density reached 70–80%, complete medium containing 4% FBS and 1% antibiotic–antimycotic was added to induce differentiation. The cells were collected from the growth medium (GM 50% and GM 100% confluency) and differentiation medium at 24, 48, 72, 96, and 120 h (referred to as DM1, DM2, DM3, DM4, and DM5, respectively). Three biological replicates were collected at each time point.

### Design of the Primers for Quantitative Real-Time PCR (qRT-PCR)

V1 software (Vazyme, Nanjing, China) combined with the stem–loop method was used to design the miRNA primers, and U6 was used as a housekeeping gene. For the genes, primers were designed using the Ensembl database combined with Premier Primer 5.0 software (Premier Biosoft International, Palo Alto, CA, United States). Meanwhile, the primer sequences for the P21, P53, myosin heavy chain (MYHC), myogenic determination 1 (MYOD1), and myogenin (MYOG) genes refer to the sequences in the work of Cai et al. (2018a). Because the HSP70 gene was very stable in embryonic muscle tissues (Wang et al., 2015), it was used as a housekeeping gene when detecting the expression level of KLF4 in embryonic and 1-day-old chickens in the present study. Meanwhile, β-actin was used as an internal control in the other time periods. These primers were synthesized by Sangon Biotech (Shanghai, China). The primers for qRT-PCR are shown in Tables 1, 2.

**TABLE 1:** MiRNA primers used for qRT-PCR.

**Primer name**	**Primer sequence (5′-3′)**	**Annealing temperature (°C)**
Gga-miR-7	F:GCGCGTGGAAGACTAGTGATT	60
	R:AGTGCAGGGTCCGAGGTATT	
U6	F:GTCACTTCTGGTGGCGGTAA	60
	R:GTTCAGTAGAGGGTCAAA	
Stem loop primer	GTCGTATCCAGTGCAGGGTCCGAG	
	GTATTCGCACTGGATACGACCAACAA	

**TABLE 2:** Gene primers used for qRT-PCR.

**Gene**	**Primer sequence (5′-3′)**	**Product size (bp)**	**Annealing temperature (°C)**	**Accession number**
KLF4	F: GCTGCGGCAAGACCTACA	113	60	XM_004949369.3
	R:TCGGGCAAACTTCCATCC			
P53	F:CCCGTAGACCACGAGCAGAT	145	60	NM_205264.1
	R:CGTCTCGGTCTCGAAGTTGA			
P21	F:GAGATGCTGAAGGAGATCAATGAG	102	60	NM_204396.1
	R:GTGGTCAGTCCGAGCCTTTT			
MYOD1	F:GCTACTACACGGAATCACCAAAT	200	60	NM_204214.2
	R:CTGGGCTCCACTGTCACTCA			
MYHC	F:CTCCTCACGCTTTGGTAA	213	60	NM_001319304.1
	R:TGATAGTCGTATGGGTTGGT			
MYOG	F:CGGAGGCTGAAGAAGGTGAA	320	60	NM_204184.1
	R:CGGTCCTCTGCCTGGTCAT			
MSTN	F:GCTCAAACAGCCTGAATCCAAT	199	60	NM_001001461.1
	R:ACATCGGGATTCCGTTGAGT			
β-actin	F:CAGCCATCTTTCTTGGGTAT	169	60	NM_205518.1
	R:CTGTGATCTCCTTCTGCATCC			
HSP70	F:TCTGCTCCTGTTGGATGTC	95	60	NM_001006685.1
	R:TGGGAATGGTGGTGTTACG			

### Extraction of RNA, cDNA Synthesis, and qRT-PCR

Total RNA was extracted from tissues and cells with Trizol (Thermo, Waltham, MA, United States) and then stored at –80°C. Next, 1.5% denaturing agarose gel electrophoresis was used to detect the integrity of the RNA, while the concentration was determined by measuring the optical density using a Nanodrop 1000c spectrophotometer (Thermo, Waltham, MA, United States). The HiScript Q RT SuperMix (Vazyme, Nanjing, China) was used for the reverse transcription of RNA into cDNA. The ChamQ SYBR qPCR Master Mix (Vazyme, Nanjing, China) was used for cDNA quantification, and the procedures were carried out in accordance with the qRT-PCR reagent instructions. cDNA synthesis for miRNA was carried out using the miRNA 1st Strand cDNA Synthesis Kit (by stem–loop) (Vazyme, Nanjing, China), followed by the miRNA Universal SYBR qPCR Master Mix (Vazyme, Nanjing, China) to detect the expression level of miR-7 in the CPMs or tissues. The qRT-PCR reactions were carried out in an Applied Biosystems^TM^ 7500 Fast Dx Real-Time PCR Instrument (ABI, Los Angeles, CA, United States). Three technical replicates were performed for each sample. The relative expression of genes and miR-7 was calculated using the 2^–ΔΔ*CT*^ method (Livak and Schmittgen, 2001).

### The Oligonucleotide Sequence Used for Overexpression, Inhibition, and Plasmid Construction

MiR-7 mimic, mimic-NC (negative control), miR-7 inhibitor, inhibitor-NC, siR-KLF4, and siR-NC were designed and synthesized by GenePharma (Shanghai, China). The oligonucleotide sequences used for the overexpression and inhibition experiments are shown in Table 3. For the overexpression of KLF4, a full-length coding domain sequence (CDS) of the KLF4 gene was synthesized by GenePharma (Shanghai, China). Using the *Bam*HI and *Eco*RI restriction sites, the synthesized sequence was inserted into the pcDNA-3.1 vector (Promega, Madison, WI, United States).

**TABLE 3:** Oligonucleotide sequences for overexpression and inhibition.

**Fragment name**	**Sequence (5′-3′)**
miR-7 mimic	UGGAAGACUAGUGAUUUUGUUG ACAAAAUCACUAGUCUUCCAUU
mimic-NC	UUCUCCGAACGUGUCACGUTT ACGUGACACGUUCGGAGAATT
miR-7 inhibitor	CAACAAAAUCACUAGUCUUCCA
inhibitor-NC	CAACAAAAUCACUAGUCUUCCA
siR-KLF4	GCCCGAUCUGAUGAACUUATT UAAGUUCAUCAGAUCGGGCTT
siR-NC	UUCUCCGAACGUGUCACGUTT ACGUGACACGUUCGGAGAATT

Using the TargetScan and miRDB online software, we found that the 3′UTR of the KLF4 mRNA has two potential binding sites for miR-7. The 3′UTR of KLF4, containing the putative binding sites for miR-7, was amplified by PCR. The pMIR-REPORT Luciferase plasmid double-digested by *Hin*dIII and *Mlu*I was recovered by the decapitation recovery kit (Axygen, Tewksbury, MA, United States), and then the amplified fragment was ligated with the linearized plasmid by the rapid seamless cloning reagent (TsingKe, Beijing, China). Finally, Sanger sequencing was used to detect whether the target fragment was successfully inserted. The mutant vectors were constructed by PCR mutagenesis. The predicted binding sites were successfully mutated from GUCUUCCA to TCATTAAC for the KLF4-1-3′UTR-MT vector, and from GUCUUCC to TCATTAA for the KLF4-2-3′-UTR-MT vector. The primers used for the vector construction are listed in Table 4.

**TABLE 4:** Primers used for vector construction.

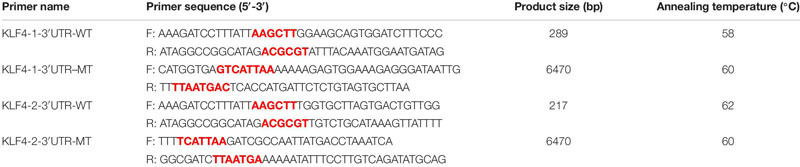

*Sequences in red represent the enzyme cutting sites.*

### Cell Transfection and Dual-Luciferase Reporter Assay

Transfection of the miR-7 mimic, miR-7 inhibitor, siR-KLF4, or pcDNA3.1-KLF4 was conducted using jetPRIME Transfection Reagent (Polyplus, Illkirch, France) following the manufacturer’s protocol. In the differentiation assay, the CPMs were transfected before the induction of the differentiation of the CPMs. 293T cells were used for the dual-luciferase reporter assay. After co-transfection, for the KLF4-3′UTR-WT (wild-type) or KLF4-3′UTR-MT (mutant-type) plasmids with the miR-7 mimic or mimic-NC, the firefly and Renilla luciferase activities were measured at 48 h post-transfection using a Dual-Luciferase Reporter Assay Kit (Vazyme, Nanjing, China) according to the instructions. Luminescence was measured using a full wavelength microplate reader (Perkin Elmer, Waltham, MA, United States) and the firefly luciferase activities were normalized to Renilla luminescence in each well.

### Flow Cytometric Analysis

The CPMs were seeded in 6-well plates and then transfected when the density reached approximately 60%. After 48 h of transfection, the cells were collected into 2 ml enzyme-free tubes. Then, 70% ethanol was added and the cells were fixed at –20°C overnight. Subsequently, the fixed cells were stained with propidium iodide (50 μg/ml; Solarbio, Beijing, China) containing 10 mg/ml RNase A (Sangon Biotech, Shanghai, China), and then incubated for 20 min at 37°C in a water bath. Flow cytometric analysis was performed on a FACSAria SORP flow cytometer (BD Company, Franklin, NJ, United States), and Modfit LT software was used to process the data.

### EdU and CCK-8 Assay

The CPMs were seeded in 24-well plates, and transfection was performed when the density reached approximately 60%. After transfection for 48 h, the CPMs were washed with ice-cold Phosphate-Buffered Saline (PBS) and fixed with 4% paraformaldehyde for 30 min. Then, the cells were treated with the 5-ethynyl-2′-deoxyuridine (EdU) assay kit (RiboBio, Beijing, China) according to the manufacturer’s instructions. Finally, cells were photographed with a fluorescence inverted microscope (Olympus, Tokyo, Japan) immediately after treatment, and the data were processed using Image-Pro Plus software. For the Cell Counting Kit-8 (CCK-8 assay), the cells were seeded in a 96-well plate and cultured with growth medium containing 15% FBS. After transfection, cell proliferation was monitored at 12, 24, 36, and 48 h using a DojinDo’ s CCK-8 kit (DojinDo, Kumamoto, Japan). Finally, absorbance at 450 nm was measured using a full wavelength microplate reader (EnSpire, Perkin Elmer, United States).

### Immunofluorescence

Chicken primary myoblasts were seeded in 12-well plates, and transfection was performed. After transfection for 12 h, the CPMs were induced to differentiate for 72 h. Then, the cells were washed with PBS (Hyclone, Logan, UT, United States) and fixed with 4% paraformaldehyde (Solarbio, Beijing, China) for 30 min. Subsequently, the cells were treated with 0.2% Triton X-100 (Solarbio, Beijing, China) for 15 min, washed with PBS, and then goat serum (Solarbio, Beijing, China) was added. The cells were incubated at 37°C for 30 min, goat serum was removed, and then primary anti-Desmin (Bioss, 1:500) was added. After incubating the cells at 4°C overnight, the cells were washed with PBST (Solarbio, Beijing, China), Cy3-labeled secondary antibody (Bioss, Beijing, China, 1:500) was added, and cells were incubated at 37°C for 1 h. Then, after washing the cells with PBST, we added 4′,6-diamidino-2-phenylindole (DAPI) staining solution (Beyotime, Shanghai, China), and incubated the cells for 2 min. Finally, an anti-fluorescence quencher (Beyotime, Shanghai, China) was added. A fluorescence inverted microscope was used to capture images, and the experimental data were processed with Image-Pro Plus software 6.0. We calculated the percentage of the total image area covered by myotubes as the myotube area. The fusion index was calculated as a percentage of nuclei inside the Desmin positive myotubes relative to the total number of nuclei.

### Western Blot Assay

The cell lysis buffer (Beyotime, Shanghai, China) was added to the CPMs. Then, the lysed cells were incubated on ice for 6 min, and the supernatant was collected after centrifugation at 12,000 rpm for 10 min at 4°C. After determination of the protein concentration, the protein samples were subjected to 10% sodium dodecyl sulfate–polyacrylamide gel electrophoresis (SDS-PAGE), and then transferred to polyvinylidene fluoride (PVDF; BIO-RAD, Hercules, CA, United States). The PVDF membrane was incubated at room temperature with 4% defatted milk powder for 1 h. Next, the primary antibodies were employed and incubated at 4°C overnight. The primary antibodies and their dilutions were as follows: MYH1 rabbit polyclonal antibody (Group Proteintech, Wuhan, China, 1:500), MYOD1 mouse monoclonal antibody (Novus, Littleton, CO, United States, 0.5 μg/ml), anti-GAPDH rabbit polyclonal antibody (BBI, Shanghai, China, 1:2000), HRP-conjugated goat anti-rabbit IgG (BBI, Shanghai, China, 1:5000). Subsequently, the PVDF membrane was washed with PBST and incubated with the secondary antibody (anti-Mouse IgG antibody; BBI, Shanghai, China, 1:2000). Next, the electrochemiluminescence (ECL) method was used to develop, and the photographs were obtained using Tanon 4200 (Tanon, Shanghai, China). The relative expression of the protein was analyzed with Quantity One software.

### Statistical Analysis

Statistical analysis was performed using SPSS19.0 software (SPSS Inc., Chicago, IL, United States). The unpaired Student’s *t*-test was used for two-group comparison analysis, and the data were considered statistically significant when *p* < 0.05 (^∗^) or *p* < 0.01 (^∗∗^). A one-way ANOVA was used for multiple-group comparison analysis. Duncan’s multiple range test was used to determine the significance, and different lowercase letters above bars indicate significant differences (*p* < 0.05). All data are presented as least squares means ± standard error of the mean (SEM).

## Results

### Expression of miR-7 and KLF4 in the Chicken Tissues

**FIGURE 1:**
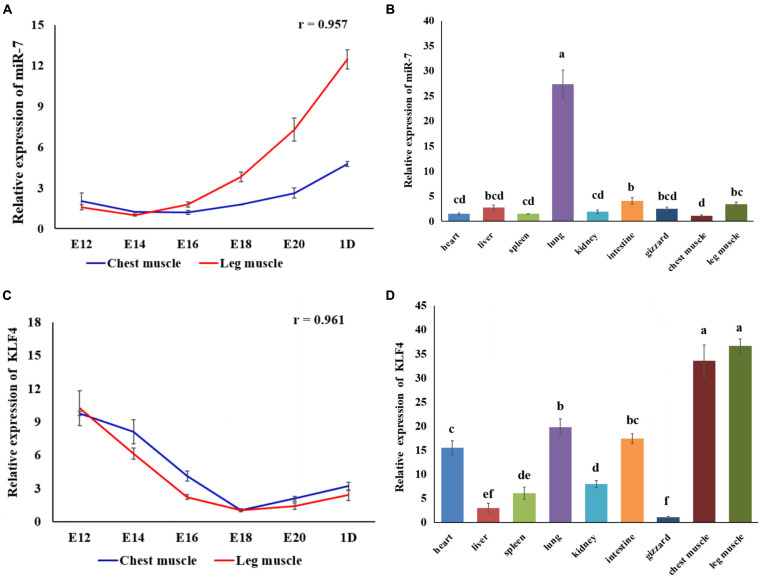
Expression of miR-7 and KLF4 gene in the tissues of Jinghai Yellow chickens. **(A)** Expression pattern of miR-7 in chest and leg muscles. **(B)** Expression profile of miR-7 in the tissues of 1-day-old chickens. **(C)** Expression pattern of the KLF4 gene in chest and leg muscles. **(D)** Expression profile of the KLF4 gene in the tissues of 1-day old chickens. E12, E14, E16, E18, E20, and 1D represent 12, 14, 16, 18, 20 embryonic days and 1-day-old, respectively. Data are presented as mean ± standard error of the mean (SEM) (*N* = 4). Different lowercase letters above bars indicate significant differences (*p* < 0.05).

Using qRT-PCR we identified the expression pattern and the tissue expression profile of miR-7 and KLF4 in Jinghai Yellow chickens. The results show that the expression level of miR-7 in the chest and leg muscles increased between E12 and day 1 (Figure 1A). The Pearson correlation analysis shows that the expression level of miR-7 in the chest and leg muscles was positively correlated (*r* = 0.957). The tissue expression profile indicates that the expression level of miR-7 in the lung was the highest, and the chest muscle had the lowest expression level. However, the expression level of miR-7 in the leg muscle was relatively higher than that in the heart, spleen, kidney, and gizzard (Figure 1B). For the KLF4 gene, the results show that the expression level of KLF4 in the chest and leg muscles decreased between E12 and day 1 (Figure 1C). The tissue expression profile shows that the expression level of KLF4 in the chest and leg muscles was the highest (Figure 1D). These results suggest that miR-7 and KLF4 might be involved in the skeletal muscle development of chickens.

### MiR-7 Inhibits the Proliferation of Chicken Primary Myoblasts

**FIGURE 2:**
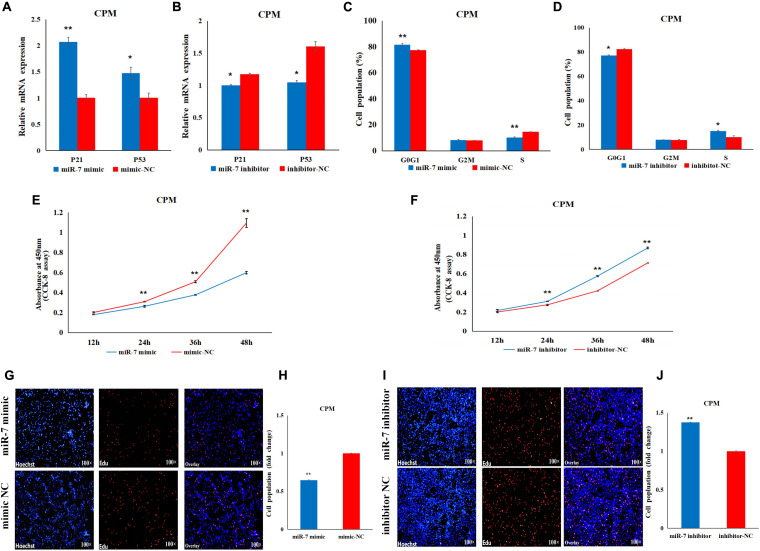
MiR-7 inhibits the proliferation of chicken primary myoblasts (CPMs). **(A,B)** Effects of the overexpression and inhibition of miR-7 on the mRNA expression of the P21 and P53 genes 48 h after the transfection. **(C,D)** The statistical results of the cell cycle analysis 48 h after the overexpression and inhibition of miR-7 in CPMs. **(E,F)** Cell growth was measured in CPMs using CCK-8 assay after overexpression and inhibition of miR-7. **(G–J)** 5-ethynyl-2′-deoxyuridine (EdU) staining of transfected CPMs and the calculation of the proliferation rate. Photomicrographs were taken using a 100× magnification. The results are shown as the mean ± SEM of three independent experiments, *p < 0.05, **p < 0.01.

In order to explore the role of miR-7 in the proliferation of CPMs, overexpression and inhibition experiments were performed, using transfection of miR-7 mimic or antisense inhibitor, respectively. P21 and P53 are two important marker genes involved in the cell cycle process. Regulating the expression of these two genes can directly affect the cell proliferation process. The results show that overexpression of miR-7 significantly increased the mRNA expression of the P21 and P53 genes (Figure 2A), while their expression levels significantly decreased when inhibiting the expression of miR-7 in CPMs (Figure 2B). The cell cycle analysis, using flow cytometry, reveals that miR-7 overexpression increased the number of cells in the G0/G1 phase, and the number of cells in the S phase was significantly reduced (Figure 2C). On the contrary, miR-7 inhibition reduced the number of cells in the G0/G1 phase, and increased the number of cells in the S phase (Figure 2D). In addition, the CCK-8 and EdU assay results show that overexpression of miR-7 significantly inhibited the proliferation of myoblasts (Figures 2E,G,H). The opposite trend occurred in CPMs treated with miR-7 inhibitor (Figures 2F,I,J). Together, these results suggest that miR-7 can inhibit the proliferation of chicken primary myoblasts.

### MiR-7 Inhibits the Differentiation of Chicken Primary Myoblasts

**FIGURE 3:**
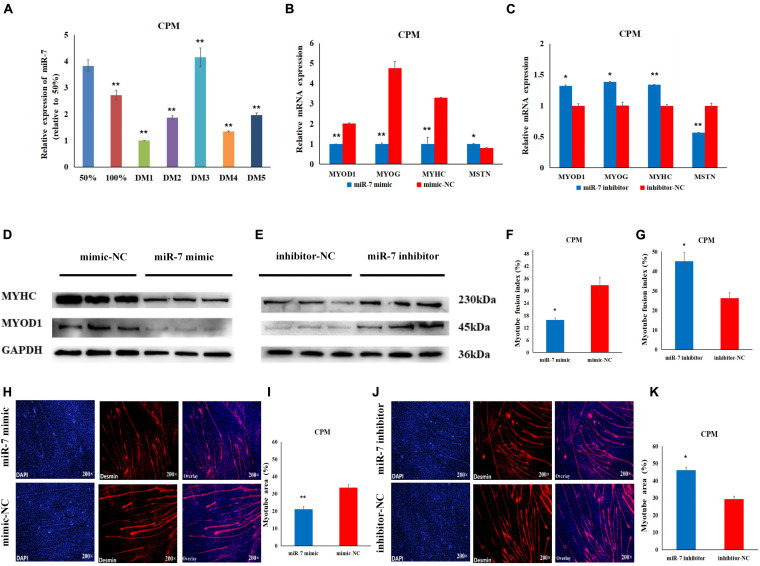
MiR-7 inhibits the differentiation of CPMs. **(A)** The expression level of miR-7 during the proliferation (50 and 100% confluency) and differentiation of CPMs. DM1, DM2, DM3, DM4, and DM5 represent CPMs which were induced to differentiate for 24, 48, 72, 96, and 120 h, respectively. **(B,C)** The mRNA expression of MYOD1, MYOG, MYHC and MSTN 48 h after the transfection of miR-7 mimic and inhibitor in CPMs. **(D,E)** The protein expression of MYHC and MYOD1 72 h after the transfection of miR-7 mimic and inhibitor in CPMs using Western Blot. **(F,G)** The myotube fusion index (%) of CPMs after the overexpression and inhibition of miR-7. **(H,J)** Connexin antibody (Desmin) staining of the myoblasts induced differentiation 72 h after transfection of the miR-7 mimic and inhibitor in CPMs. **(I,K)** The myotube area (%) of CPMs after the overexpression and inhibition of miR-7. Photomicrographs were taken using a 200× magnification. The results are shown as the mean ± SEM of three independent experiments, *p < 0.05, **p < 0.01.

In order to explore the role of miR-7 in the differentiation of myoblasts, differentiation was induced in the CPMs, by switching cells into low-serum-containing medium when they had reached 70–80% confluency. qRT-PCR experiments show that miR-7 had a relatively low expression when the CPMs started differentiation (Figure 3A). Overexpression of miR-7 significantly down-regulated mRNA expression of the three differentiation marker genes [i.e., myogenic determination 1 (MYOD1), myogenin (MYOG), and myosin heavy chain (MYHC)] (*p* < 0.01), and significantly up-regulated mRNA expression of the myostatin (MSTN) gene (*p* < 0.05), while inhibition of miR-7 had the opposite trend (Figures 3B,C). The Western Blot experiments indicate that the MYHC and MYOD1 proteins were down-regulated after miR-7 overexpression, while they were increased after miR-7 inhibition (Figures 3D,E). Moreover, the immunofluorescence staining shows that miR-7 overexpression significantly reduced the fusion index and the area of myotubes (Figures 3F,H,I), while inhibition of miR-7 was able to significantly promote the formation of myotubes (Figures 3G,J,K). Overall, the results suggest that miR-7 inhibits the differentiation of chicken primary myoblasts.

### KLF4 Is a Target Gene of MiR-7

**FIGURE 4:**
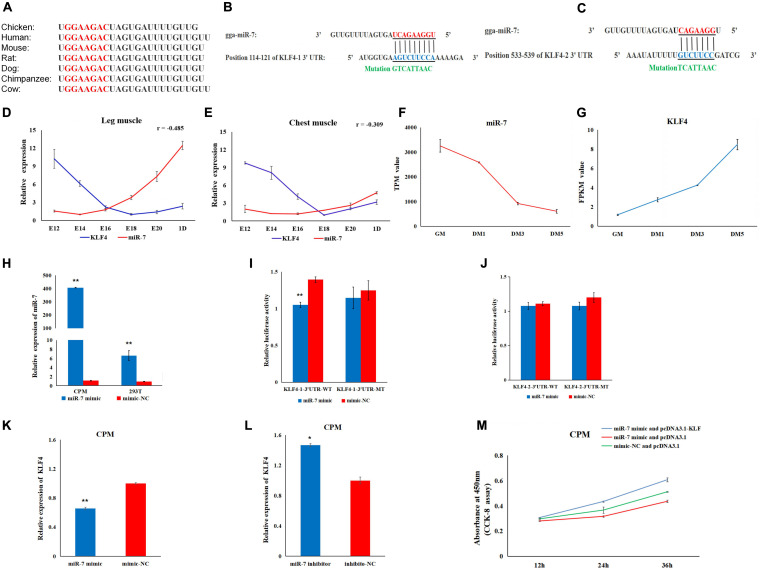
KLF4 is a target gene of miR-7. **(A)** The seed sequence of miR-7 is highly conserved among vertebrates. **(B,C)** The potential binding sites for miR-7 in the 3′untranslated region (3′UTR) of KLF4 (KLF4-1 3′UTR and KLF4-2 3′UTR). **(D,E)** The expression trends of miR-7 and the KLF4 gene in chest and leg muscles. E12, E14, E16, E18, E20, and 1D represent 12, 14, 16, 18, 20 embryonic days and 1-day-old of Jinghai Yellow chickens, respectively. **(F,G)** The expression levels of miR-7 and the KLF4 gene during proliferation and differentiation stages using RNA-seq data. GM represents CPMs in the proliferation stage (100% confluence). DM1, DM3, and DM5 represent CPMs which were induced to differentiate for 24, 72 and 120 h, respectively. TPM is the abbreviation of Transcripts Per Million, and FPKM is the abbreviation of Fragments Per Kilobase per Million **(H)** Overexpression efficiency 48 h after transfection of the miR-7 mimic in CPMs and 293T cells. **(I,J)** Luciferase assays were performed by co-transfection of wild-type or mutant KLF4 3′UTR with a miR-7 mimic or mimic-NC. **(K,L)** The mRNA expression level of KLF4 were detected 48 h after being transfected with the miR-7 mimic or inhibitor in CPMs. **(M)** The results of the rescue experiment in CPMs using CCK-8 assay. The results are shown as the mean ± SEM of three independent experiments, *p < 0.05, **p < 0.01.

To explore the regulatory mechanism of miR-7 in the proliferation and differentiation of CMPs, we combined the miR-7 target prediction and the RNA-Seq results. The seed sequence in miR-7 is conserved among vertebrates (Figure 4A). Using the TargetScan and miRDB online software, two potential binding sites for miR-7 in 3′UTR of KLF4 were found (Figures 4B,C). Our previous RNA-seq data shows that miR-7 was relatively highly expressed in the proliferation phase of CPMs, but its expression level decreased significantly after the differentiation of CPMs (Supplementary Table S1 and Figure 4F). At the same time, the expression trend of the KLF4 gene was the opposite (Supplementary Table S1 and Figure 4G). According to the Pearson correlation coefficient, the expression level of miR-7 and KLF4 detected by qRT-PCR was negatively related in both the chest and leg muscles (Figures 4D,E).

To verify the possible target relationship between miR-7 and KLF4, a dual-luciferase reporter assay was carried out (Figures 4I,J). When CPMs and 293T cells were transfected with miR-7 mimic, respectively, the expression level of miR-7 was significantly increased compared to the NC group (*P* < 0.01) (Figure 4H). Compared to the NC group, the miR-7 mimic was able to reduce the luciferase activity after being co-transfected with KLF4-1-3′UTR-WT vector, whereas no significant difference was observed in the mutant group (KLF4-1-3′UTR-MT) (Figure 4I). Meanwhile, after the overexpression of miR-7 in the CPMs, the mRNA level of KLF4 was significantly decreased, and then up-regulated after inhibition of miR-7 (Figures 4K,L). To further evaluate the role of miR-7 in the functional effects of KLF4, we co-transfected in CPMs with miR-7 mimic and pcDNA3.1-KLF4, miR-7 mimic and pcDNA3.1, or mimic-NC and pcDNA3.1, respectively. The results of the CCK-8 assay show that the simultaneous overexpression of miR-7 and KLF4 promoted proliferation (Figure 4M). Taken together, these results indicate that there is a direct target relationship between miR-7 and KLF4.

### KLF4 Promotes the Proliferation of Chicken Primary Myoblasts

**FIGURE 5:**
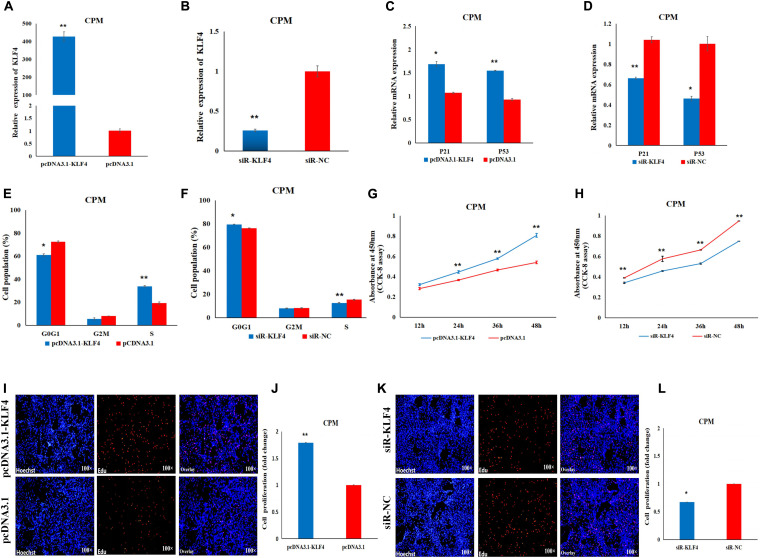
KLF4 promotes the proliferation of CPMs. **(A)** The expression level of KLF4 after its 48 h overexpression in CPMs. **(B)** The expression level of the KLF4 gene after its 48 h inhibition in CPMs. **(C,D)** The P21 and P53 mRNA expression levels 48 h after the overexpression and inhibition of the KLF4 gene in CPMs. **(E,F)** Cell cycle analysis after KLF4 gene overexpression and inhibition in CPMs. **(G,H)** Proliferation curve within 48 h after the overexpression and inhibition of the KLF4 gene in CPMs. **(I,K)** EdU proliferation assays for CPMs after overexpression and inhibition of the KLF4 gene. **(J,L)** The fold change of the proliferation rates after overexpression and inhibition of the KLF4 gene. Photomicrographs were taken using a 100× magnification. The results are shown as the mean ± SEM of three independent experiments, *p < 0.05, **p < 0.01.

In order to determine the role of KLF4 in the proliferation and differentiation of CPMs, we constructed and validated an overexpression vector (pcDNA3.1-KLF4) and a siRNA (siR-KLF4) (Figures 5A,B). In the CPMs, qRT-PCR shows that overexpression of KLF4 was able to significantly up-regulate the expression level of the P53 and P21 genes, whereas inhibition of KLF4 was able to decrease the expression level of these two genes (Figures 5C,D). In the cell cycle analysis using flow cytometry, overexpression of KLF4 resulted in a significant decrease in the number of cells in the G0/G1 phase, and a significant increase in the number of cells in the S phase (Figure 5E). The opposite trend occurred in CPMs treated with siR-KLF4 (Figure 5F). In addition, the results of the CCK-8 and EdU assays show that the overexpression of the KLF4 gene significantly promoted the proliferation of myoblasts (Figures 5G,I,J). Conversely, inhibition of KLF4 significantly repressed the proliferation of CPMs (Figures 5H,K,L). These results suggest that KLF4 can promote the proliferation of chicken primary myoblasts.

### KLF4 Promotes the Differentiation of Chicken Primary Myoblasts

**FIGURE 6:**
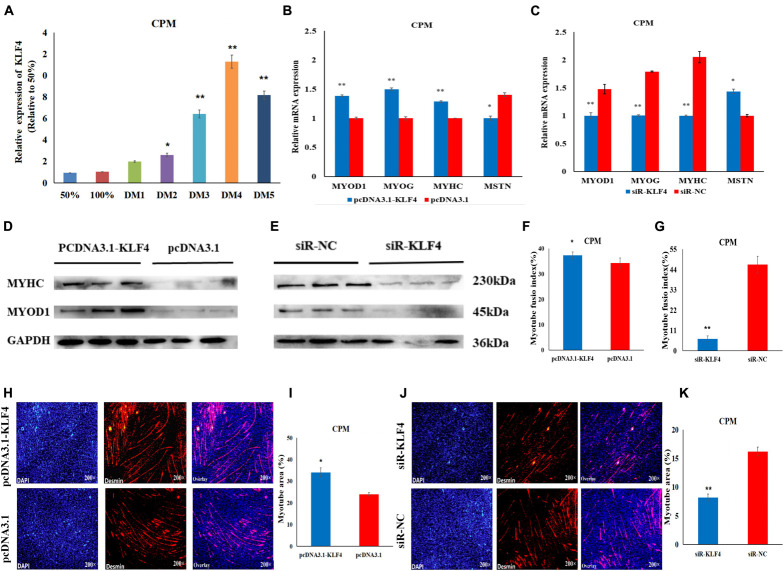
KLF4 promotes the differentiation of CPMs. **(A)** The expression level of KLF4 during the proliferation (50 and 100% confluency) and differentiation of CPMs. DM1, DM2, DM3, DM4, and DM5 represent CPMs which were induced to differentiate for 24, 48, 72, 96, and 120 h, respectively. **(B,C)** The mRNA expression of MYOD1, MYOG, MYHC and MSTN 48 h after the overexpression and inhibition of the KLF4 gene in CPMs. **(D,E)** The protein expression of MYHC and MYOD1 72 h after the overexpression and inhibition of the KLF4 gene in CPMs using Western Blot. **(F,G)** The myotube fusion index (%) of the CPMs after KLF4 overexpression and inhibition. **(H,J)** Immunofluorescence analysis of Desmin-staining cells after KLF4 overexpression and inhibition. **(I,K)** The myotube area (%) of the CPMs after KLF4 overexpression and inhibition. Photomicrographs were taken using a 200× magnification. The results are shown as the mean ± SEM of three independent experiments, *p < 0.05, **p < 0.01.

To further investigate the role of KLF4 in the differentiation of chicken myoblasts, the CPMs were induced to differentiate *in vitro* when they had reached 70–80% confluency. The qRT-PCR results show that the expression level of KLF4 increased gradually during differentiation (Figure 6A). Meanwhile, the overexpression of KLF4 significantly increased the mRNA expression of the three differentiation marker genes (i.e., MYOD1, MYOG, and MYHC), and inhibited the mRNA expression of the MSTN gene (Figure 6B). Meanwhile, the inhibition of KLF4 had opposite effects on the mRNA expression of these genes (Figure 6C). Moreover, Western Blot experiments show that the protein expression levels of MYHC and MYOD1 were up-regulated after the overexpression of KLF4 (Figure 6D), and down-regulated after the inhibition of KLF4 (Figure 6E). The results of the immunofluorescence staining show that the differentiation of myoblasts was significantly increased after KLF4 overexpression (Figures 6F,H,I). Conversely, the inhibition of KLF4 significantly decreased the fusion index and the myotube area of myotubes (Figures 6G,J,K). These results demonstrate that KLF4 can promote the differentiation of chicken primary myoblasts.

## Discussion

Studies have shown that miR-7 is involved in growth and development, as well as in diseases (Li et al., 2018b; Matoušková et al., 2018; Zeng et al., 2018). However, the way in which miR-7 regulates the proliferation and differentiation of chicken myoblasts remains unknown. It is well known that miRNAs bind to sites located in the 3′untranslated regions (3′UTRs) of mRNAs to down-regulate their expression (Gu et al., 2009). Dong et al. (2019) showed that miR-7 has two binding sites in 3′UTR of the KLF4 gene in human colorectal cancer cells. We further examined the target relationship between miR-7 and KLF4 by dual-luciferase assay in 293T cells. The results show that miR-7 functions by binding the first site of KLF4 (KLF4-1-3′UTR-WT). Moreover, the rescue experiment in CPMs also demonstrated that KLF4 was a target gene of miR-7 in chickens.

In this study, the *in vitro* experiment shows that miR-7 had a relatively low expression when the CPMs started to differentiate, while the expression level of KLF4 increased gradually during differentiation. Using our previous RNA-seq data (Supplementary Table S1), the results also show that miR-7 was relatively highly expressed in the proliferation phase, and its expression level decreased significantly after the differentiation of CPMs, while the KLF4 gene had the opposite regularity in expression (Figures 4F,G). The qRT-PCR results were consistent with the previous RNA-seq results. However, in the *in vivo* experiment, our results show that miR-7 expression increased between E12 and day 1 in chest and leg muscles, when muscle growth and differentiation occurred. While the expression of miR-7 decreased when the CPMs started to differentiate. Cai et al. (2018a) found that miR-16-5p can inhibit the proliferation and differentiation of chicken myoblasts, while the expression of miR-16-5p increased between E10 and E19 in chest muscle, and miR-16-5p expression in CPMs decreased during differentiation. In another paper, it was reported that miR-205a can promote the differentiation of chicken myoblasts, while the expression of miR-205a decreased between E12 and E20 in leg muscle (Wang et al., 2018b). These results are consistent with our results. Our research also showed that KLF4 expression decreased between E12 and day 1 in chest and leg muscles, while KLF4 expression increased in CPMs during differentiation. We deduced that the expression of KLF4 was regulated by miR-7. As miR-7 expression increased, KLF4 expression decreased accordingly. Whereas, both miR-7 and the target gene KLF4 had conflicting expression profiles in skeletal muscle *in vivo* versus the CPMs in culture. This may indicate that miR-7/KLF4 plays different roles in skeletal muscle versus CPMs.

In the proliferation experiment, we found that the mRNA expression levels of the P21 and P53 genes were increased after the overexpression of miR-7, and flow cytometry analysis indicated that the overexpression of miR-7 blocked the cell proliferation process, while interference with miR-7 had the opposite effect. Previous studies have shown that both the P21 and P53 genes have the effect of inducing apoptosis to arrest the cell cycle (Speidel, 2010; Liu et al., 2017), and P53-mediated cell cycle arrest is mainly caused by P53-dependent transcription of P21 (Massagué, 2004). Cai et al. (2018a) reported that miR-16-5p can increase the expression of P21, and can repress the proliferation of chicken myoblasts. In our study, miR-7 may have affected the cell distribution in the cell cycle and ultimately the proliferation of CPMs by regulating the expression of the P21 and P53 genes. Through a series of *in vitro* experiments, the results of the CCK-8 and EdU assays indicated that the overexpression of miR-7 inhibited the proliferation of CPMs, and interference with miR-7 was able to promote the proliferation of CPMs.

The KLF4 gene, called the epidermal zinc finger factor, is a zinc-containing finger structure protein and is a member of the KLF family (Elemento, 2016; Tian et al., 2016). Members of this family belong to the family of eukaryotic transcriptional factors with a zinc-containing finger structure, and they are mainly involved in the regulation of life activities such as cell growth, differentiation, and apoptosis (Zhang et al., 2017; Yang et al., 2018; Ma et al., 2019). Meanwhile, the pathway for the chicken KLF4 gene has not been elucidated (Gene ID: 770254, NCBI). In the present research, after the overexpression of the KLF4 gene, the mRNA expression levels of the P21 and P53 genes increased, and the mRNA expression levels of the P21 and P53 genes decreased after interference with the KLF4 gene. Comparing our results with the results reported for the SESN1 gene by Cai et al. (2018a), KLF4 and SESN1 had opposite effects on the expression of the P21 and P53 genes. However, the internal causes need to be further explored. Nevertheless, flow cytometry analysis showed that the KLF4 gene was able to affect the cell cycle of CMPs. The CCK-8 and EdU assays showed that the overexpression of KLF4 was able to promote the proliferation of CPMs. Conversely, interference with the KLF4 gene was able to inhibit the proliferation of CPMs.

In the induction differentiation assay, the results showed that the overexpression of miR-7 reduced the mRNA or protein expression levels of the differentiation marker genes (i.e., MYHC, MYOD1, and MYOG), while it increased the mRNA expression level of the MSTN gene. Luo et al. (2014) found that overexpression of MiR-203 was also able to reduce the expression of MYHC, MYOD1, and MYOG. It is well known that MSTN is a negative regulator during skeletal muscle development (Mcpherron et al., 1997). The high expression of MSTN in our research also indicated that the differentiation of CPMs was inhibited. Conversely, interference with miR-7 had the opposite effect on the expression of the detected genes and on the differentiation of myotubes. These results suggest that miR-7 might play a negative regulatory role in the differentiation of CPMs. Some studies have found that a novel circular RNA acting as a miR-7 inhibitor promoted the osteogenic differentiation of periodontal ligament stem cells (PDLSCs), which indicates that miR-7 could inhibit the osteogenic differentiation of PDLSCs (Li et al., 2018b).

Chen et al. (2018) found that steroid receptor co-activator 3 (SRC-3) protects the intestines from dextran sulfate sodium (DSS)-induced colitis by promoting goblet cell differentiation through enhancement of KLF4 expression. In our research, the overexpression of KLF4 significantly promoted mRNA expression of the three differentiation marker genes (i.e., MYHC, MYOD1, and MYOG), as well as the protein expression of MYHC and MYOD1 and differentiation of CPMs. In contrast, interference with the KLF4 gene had the opposite effect. Wang et al. (2020) showed that KLF4 might be a target for the therapy of pulmonary fibrosis by inhibiting myofibroblast differentiation of lung-resident mesenchymal stem cells (LR-MSCs). Our results indicate that the KLF4 gene can promote the differentiation of CPMs and can affect the development of skeletal muscle in chickens.

## Conclusion

In conclusion, miR-7 can inhibit the proliferation and differentiation of CPMs by targeting the KLF4 gene. KLF4 plays a positive role in the proliferation and differentiation of CPMs. Our findings elucidated the mechanism by which miR-7 regulates the proliferation and differentiation of CPMs. These new findings provide a theoretical basis for understanding the molecular mechanisms of the proliferation and differentiation of chicken primary myoblasts.

## Data Availability Statement

The datasets presented in this study can be found in online repositories. The names of the repository/repositories and accession number(s) can be found in the article/ Supplementary Material.

## Ethics Statement

The animal study was reviewed and approved by the Animal Ethics Committee of Yangzhou University.

## Author Contributions

FC, PW, MH, and GZ were responsible for the cell culture experiments and the analysis of the experimental data. TL and YD were responsible for the analysis of RNA-Seq data. GZ and FC were responsible for the writing of the manuscript and the modification of the grammatical syntax. FC, TZ, HS, and XY were responsible for tissue collection and RNA extraction. JW, GD, and KX provided the consumables required for the experiment, as well as the experimental instruments. GZ carried out the design of the research. All authors finally read and approved the revised draft.

## Conflict of Interest

HS was employed by the company Jiangsu Jinghai Poultry Group Co., Ltd.

The remaining authors declare that the research was conducted in the absence of any commercial or financial relationships that could be construed as a potential conflict of interest.
